# Rupture of Non-communicating Rudimentary Horn of Uterus at 12 Weeks’ Gestation

**DOI:** 10.7759/cureus.7191

**Published:** 2020-03-06

**Authors:** Mariette Bruand, Thibault Thubert, Norbert Winer, Pauline Gueudry, Vincent Dochez

**Affiliations:** 1 Obstetrics and Gynecology, Centre Hospitalier Universitaire de Nantes, Nantes, FRA

**Keywords:** rudimentary uterine horn, pregnancy, unicornuate uterus, mullerian anomaly, uterine rupture

## Abstract

The rudimentary horn of uterus is an extremely rare malformation and potentially serious obstetric entity, threatening maternal and fetal outcome. Diagnostic sonography of early pregnancy in a non-communicating rudimentary horn is difficult but important. We report a case of ruptured non-communicating rudimentary horn with unicornuate uterus at 12 weeks’ gestation, where diagnosis is made before surgery. Excision of the rudimentary horn and ipsilateral salpingectomy (to prevent a further ectopic tubal gestation), conserving the ovary, is the recommended surgical procedure for patients desiring to maintain their fertility potential. The subsequent obstetric prognosis is reassuring. Diagnostic imaging examinations of the reproductive system after this treatment showed no negative effect from surgery on subsequent fertility and there was no reported case of uterine rupture during subsequent pregnancy in the remaining unicornuate uterus after rudimentary horn excision. Future pregnancies will require extremely close monitoring and a caesarean section is highly recommended.

## Introduction

Unicornuate uterus with a rudimentary horn results from arrested development of one of the Mullerian ducts. The majority of patients are asymptomatic but this rudimentary horn may contain a cavity with endometrium and be the seat of implantation of a pregnancy [1,2].

The incidence of rudimentary horn pregnancy is estimated between 1/100,000 and 1/140,000 pregnancy. In most cases, the natural history of this atypical pregnancy is rupture of the pregnant horn during the first or second trimester, resulting in life-threatening heavy bleeding, and diagnosis is made during surgery for maternal rescue [1,3,4].

The ultrasound diagnosis of a pregnancy in a malformed uterus, in particular in a rudimentary horn, is important given the severity of the situation, but remains difficult. The achievement of the ultrasound by experienced operators seems essential.

A case is presented demonstrating a non-communicating rudimentary horn connected to a unicornuate uterus, where diagnosis was made prior to surgery.

## Case presentation

An 18-year-old healthy woman, gravida 2, para 0, with a history of abortion by vacuum extraction, presented to the emergency department with abdominal pain in early pregnancy.

She had no previous medical or surgical history of relevance. According to her dates she was thought to be 12 weeks pregnant; no ultrasound had yet been performed.

**Figure 1:**
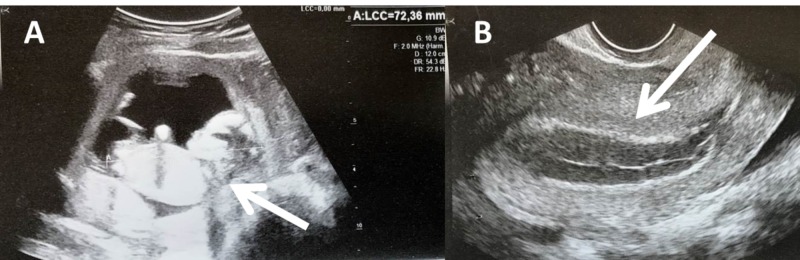
Ultrasound images. (A) First abdominal ultrasound: intrauterine pregnancy (Arrow) with myometrium. (B) Transvaginal ultrasound: vacuity uterine with gravid endometrium (Arrow).

The patient was hemodynamically stable, with no tachycardia or hypotension. The first examination was reassuring, as there was no bleeding, and a pelvic ultrasound showed an intrauterine pregnancy with an embryo measuring 72 mm, biometric indices were consistent with the menstrual age, and cardiac activity was visualized (Figure 1). The patient was discharged, but she was reevaluated the next day for worsening pain with vomiting.

A new pelvic ultrasound was performed that showed an embryo with cardiac activity, inside a uterine cavity but the myometrium looked fine. No intraperitoneal effusion was visualized. A unicorn uterus was visualized in the left, and pregnancy inside another cavity. No 3D ultrasound had been performed before, and the uterine malformation was not previously labeled.

**Figure 2:**
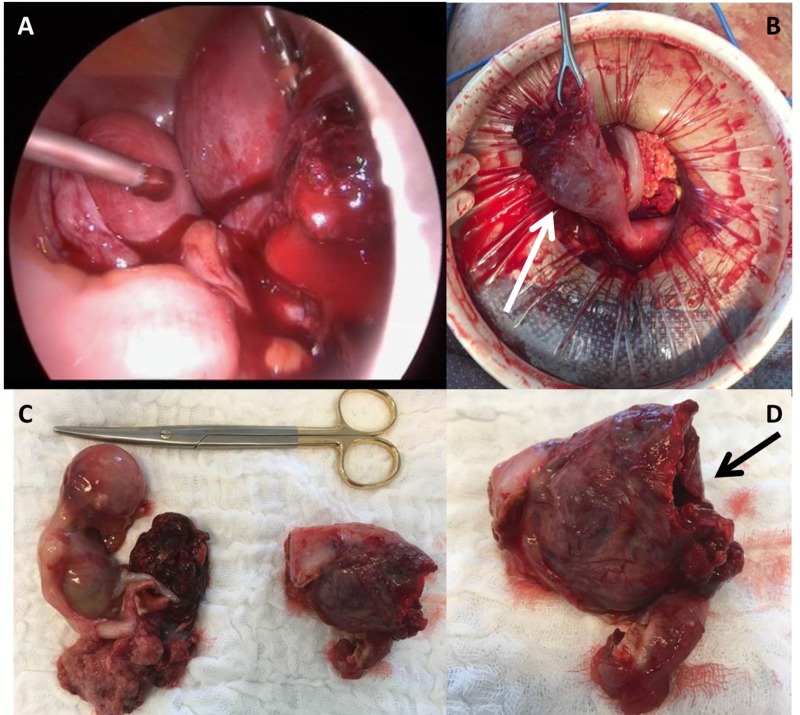
Intraoperative images. (A) Intraoperative laparoscopic image. (B) Rupture of a gravid rudimentary uterine horn in the right unicornuate uterus (Arrow) with the healthy fallopian tube in the left. (C) Histopathological examination with rupture of the right rudimentary horn, ipsilateral salpingectomy and fetus with placenta. (D) Histopathological examination with rupture of the right rudimentary horn (Arrow).

Given that the patient’s pain progressed, a decision was made to perform a diagnostic laparoscopy immediately. Laparoscopy revealed rupture of a gravid rudimentary uterine horn in the right side, causing excessive intraperitoneal bleeding (Figure 2).

Due to an enlarging gravid uterus, it became necessary to perform a laparotomy. Excision of the right rudimentary horn and ipsilateral salpingectomy was performed with a LigaSureTM device. The entire cavity was removed with the right tube. Hemostasis was also provided by several separate points of a 0 braided suture on the area of communication with the remaining uterine horn. One liter of blood was aspirated from the abdominal cavity. Four units of blood were transfused intra- and postoperatively. Her post-op recovery was uneventful. She was discharged on post-op day 3.

## Discussion

According to the European Society of Human Reproduction and Embryology (ESHRE) classification, unicornuate uterus is classed U4a [5]. It is reported that the rudimentary horn is preferentially situated on the right (62%), as it was in the above case, because the left Müller's canal progresses more caudally than the right [1,3].

Sonographic diagnosis of this malformation is challenging due to a limited field of view compared to other diagnostic imaging modalities and lateral deviation of the rudimentary horn [6]. Due to space limitation, 90% of such pregnancies end by rupture of the rudimentary horn. Most gravid rudimentary horn ruptures occur between 10 and 20 gestational weeks as a consequence of progressive myometrial thinning with advancing gestational age [1,7,8].

In the above case the vacuity uterine was clearly visualized during the transvaginal examination and diagnosis was established prior to surgery. In the majority of cases published, pelvic ultrasound led to a misdiagnosis of intrauterine pregnancy (because of the presence of myometrium in the rudimentary horn) and diagnosis was made during surgery performed for hemorrhagic shock [4].

When a rudimentary uterine horn ruptures, surgery essentially facilitates diagnosis and enables the definitive treatment. Excision of the rudimentary horn and ipsilateral salpingectomy (to prevent a further ectopic tubal gestation), with the intention of conserving the ovary, is the recommended surgical procedure for patients desiring to maintain their fertility potential. In recent years, several cases were treated by laparoscopy [9,10]. Laparoscopy can be safe and efﬁcient in early unruptured cases, with low complication rate especially in cases where there has been evaluation of the rudimentary horn and any associated urological anomalies were done before surgery [4,8,10]. In the above case, because of a large gravid uterus, and significant bleeding, laparoscopic findings warranted conversion to exploratory laparotomy for definitive surgical management.

The subsequent obstetric prognosis is reassuring. Diagnostic imaging examinations of the reproductive system after this treatment showed no negative effect from surgery on subsequent fertility and there was no reported case of uterine rupture during subsequent pregnancy in the remaining unicornuate uterus after rudimentary horn excision [11].

Also, urinary tract anomalies are common in women with unicornuate uterus. In a review of literature, Jayasinghe et al. found renal anomalies in 59 (36%) of 165 cases of rudimentary horn [12]. The need to evaluate the urinary tract in these patients must be stressed.

## Conclusions

Pregnancy in a rudimentary uterine horn is a rare form of ectopic pregnancy, which can be difficult to diagnose clinically or even with diagnostic imaging. Early diagnosis of this uterine abnormality is essential, before pregnancy if possible or in the first trimester. Therefore, location of pregnancy and detection of uterine malformations is essential. Understanding the sonographic appearance of early pregnancy in a non-communicating rudimentary horn is important and can be life-saving.
